# Transcription factor activity divergence across muscle atrophy conditions: A comparative analysis of spaceflight, aging, and disuse using decoupleR

**DOI:** 10.1371/journal.pone.0359659

**Published:** 2026-10-01

**Authors:** Niraj Shah

**Affiliations:** Independent Researcher, Cerritos, California, United States of America; Universitatsklinikum Hamburg-Eppendorf, GERMANY

## Abstract

Skeletal muscle atrophy arises from heterogeneous insults including spaceflight, aging, and immobilization. Prior studies have catalogued differentially expressed genes (DEGs) in each context, but the transcription factors (TFs) coordinating these programs remain inconsistently characterized across conditions, and gene-level overlap between conditions is small. Three mouse transcriptomic datasets were analyzed: NASA OSD-576 (Rodent Research-23) tibialis anterior muscle from spaceflight versus ground control animals; GSE145480 gastrocnemius muscle from 28-month-old versus 8-month-old mice as a sarcopenia model; and GSE273092 gastrocnemius muscle from mice subjected to ten days of hindlimb unloading versus weight-bearing controls as a disuse atrophy model. For each, gene-level Wald statistics from PyDESeq2 were used to infer TF activity with decoupleR’s univariate linear model (ULM) against the CollecTRI mouse regulon (43,226 TF-target interactions, 1,165 TFs). TFs were classified as pan-atrophy, partial, or condition-specific based on |NES| ≥ 1.5 in 3, 2, or 1 conditions. Of 732 TFs scored, 47 (6.4%) were pan-atrophy, 161 (22.0%) partial, and 295 (40.3%) condition-specific. Pearson correlation of TF activity profiles was highest between spaceflight and disuse atrophy (r = 0.4573) and lowest between spaceflight and sarcopenia (r = 0.1459). Pan-atrophy regulators included the glucocorticoid receptor Nr3c1, Foxo1, the chromatin remodeller Smarca4, and the stress regulators Pml and Ing4; Stat1 crossed the threshold but showed a sign-discordant pattern, repressed in spaceflight and disuse but activated in sarcopenia. Spaceflight showed repression of Hsf1/Hsf2/Hsf4 and activation of Hdac7; sarcopenia uniquely activated Spi1, Stat5a, and Irf2; disuse atrophy uniquely activated Mlxipl and Ovol1. This comparative transcriptomic analysis reveals a small but biologically coherent set of pan-atrophy regulators dominated by glucocorticoid and FoxO signaling, alongside a much larger pool of condition-specific regulators encoding the stressor of origin, narrowing the candidate regulator list warranting further investigation for relevance to muscle wasting broadly and to spaceflight specifically.

## Introduction

Skeletal muscle accounts for about 40% of body mass in healthy adults and is the main organ of locomotion, postural support, thermogenesis, and amino acid metabolism [1]. Loss of muscle mass and function, broadly referred to as muscle atrophy, accompanies aging, immobilization, denervation, cancer cachexia, glucocorticoid excess, and exposure to microgravity [2,3]. Despite arising from mechanistically distinct stressors, these atrophy contexts are typically studied individually, and it remains undetermined whether they share transcriptional regulatory programs or represent independent wasting pathways, a question with direct clinical and translational relevance, since a shared regulatory core would identify therapeutic targets, while stressor-specific programs would instead need context specific interventions.

This question carries substantial clinical weight for each of the three conditions considered here. Sarcopenia (aging-associated muscle atrophy) affects an estimated 5–13% of adults aged 60–70 and more than 50% of those over 80, contributing to falls, frailty, loss of independence, and increased mortality for any reason [4,5]. Disuse atrophy from bed rest, casting, or limb unloading can drive losses of 1–2% of leg muscle mass per day during the first weeks of immobilization and is a leading complication of ICU stays and orthopedic recovery [6,7]. Spaceflight imposes a combination of mechanical unloading, fluid shifts, ionizing radiation, and circadian disruption that causes atrophy of postural muscles in astronauts within weeks of orbit, and remains a medical risk for long-term exploration missions [8,9]. Whether interventions developed for one of these conditions could be expected to be solutions to the others depends directly on the degree of shared regulatory biology between them, which is the main problem this study addresses.

Mechanistically, muscle atrophy converges on increased proteolysis through the ubiquitin-proteasome and autophagy-lysosome pathways and on suppressed protein synthesis. Sandri and colleagues established that the Forkhead box O (FoxO) family directly transactivates the muscle-specific E3 ubiquitin ligases Atrogin-1/Fbxo32 and MuRF1/Trim63, required for atrophy in fasting, immobilization, denervation, and glucocorticoid models [2,10]. Parallel work identified canonical NF-κB signaling, the integrated stress response (ATF4, ATF3, DDIT3), TGF-β/Smad signaling, p53, and glucocorticoid receptor signaling as additional drivers of atrophy-related transcription, counterbalanced by IGF1-Akt-mTOR signaling, which promotes protein synthesis and antagonizes FoxO-driven proteolysis [11–14]. However, the relative contribution of each regulatory axis depends on atrophy contexts, and most experimental studies have characterized only one stressor in isolation, leaving the cross-condition question largely untested at the mechanistic level.

The handful of comparative transcriptomic studies that do exist have asked whether different atrophy stimuli converge on common gene expression programs, rather than common regulatory programs, which is an important distinction, since shared downstream gene expression can arise from entirely different upstream regulators, and conversely, a shared regulator can drive divergent downstream genes across tissue contexts. Studies comparing immobilization, denervation, fasting, glucocorticoid treatment, and aging have identified small overlapping atrophy-gene cores (typically 50–250 genes such as Atrogin-1, MuRF1, Cathepsin L, and Foxo3), but have also found extensive context-specific transcription [15,16]. Spaceflight datasets have similarly revealed gene-level overlaps with hindlimb unloading and with sarcopenia that are smaller than expected from shared phenotypes [8,17]. This modest gene-level overlap means there are two competing explanations that prior studies have been unable to distinguish: either the wasting program is redundantly encoded at the gene level (many different genes achieving the same functional outcome), or convergence exists but is only detectable at a higher level of regulation, specifically, at the level of the transcription factors (TFs) that coordinate expression, rather than the downstream genes themselves [18]. Resolving this requires shifting the unit of comparison from individual genes to inferred TF activity, which is the approach taken here.

Recent advances in network-based statistics make this shift practical. TF activity can now be estimated directly from differential expression by aggregating the behavior of curated target-gene sets, without requiring gene-level overlap between conditions. The decoupleR framework provides a unified interface to a family of such methods, including the univariate linear model (ULM) used here, and has been validated for both bulk and single-cell RNA-seq across multiple species [18,19]. The CollecTRI regulon is the most comprehensive resource of literature-supported TF-target interactions for human and mouse, extending the earlier DoRothEA collection with additional confidence tiering and signed mode-of-regulation [20,21]. Together, decoupleR and CollecTRI shift comparative transcriptomics from descriptive gene-list overlap to mechanistically interpretable activity scores per TF, which is exactly the tool needed to test whether spaceflight, sarcopenia, and disuse atrophy share a regulatory core despite their limited gene-level overlap.

Based on available literature, no prior study has compared muscle atrophy conditions at the level of TF activity. Using the NASA GeneLab OSD-576 (RR-23) spaceflight dataset [22] with two publicly available datasets covering aging-associated sarcopenia (GSE145480) and hindlimb unloading-induced disuse atrophy (GSE273092), a 732 TF × 3 condition activity matrix was computed that formally classifies TFs as pan-atrophy, partial, or condition-specific, and directly test whether a coherent pan-atrophy regulatory program exists that is obscured at the level of individual genes, which resolves the ambiguity left by prior gene-level comparisons.

## Methods

### Dataset acquisition and preprocessing

Three publicly available mouse skeletal-muscle transcriptomic datasets represent canonical muscle atrophy stressors. Spaceflight: NASA OSD-576 (Rodent Research-23, RR-23) provided RNA-seq of tibialis anterior muscle from 9 female C57BL/6J mice flown to the International Space Station for ~37 days (FLT) and 9 ground-control animals (GC) housed in identical habitats at the NASA Kennedy Space Center [23]. Counts had been pre-quantified upstream using the GeneLab consensus pipeline (STAR alignment to GRCm38 followed by RSEM quantification) and were re-imported as an 18 × 12,633 sample-by-gene integer matrix (expr_df_factors.csv) with FLT (n = 9) versus GC (n = 9). Sarcopenia: GSE145480 [24] provided polyA RNA-seq of gastrocnemius muscle from female wild-type C57BL/6JRj mice across an aging time-course (8, 18, 22, 24, 26, and 28 months). To define a binary sarcopenia signature, the oldest 28-month animals (n = 9) were contrasted against the youngest 8-month animals (n = 8). GSE145480 includes four intermediate ages (18, 22, 24, and 26 months) in addition to the 8- and 28-month extremes used here. The extremes were chosen specifically to maximize the transcriptional contrast available for a binary DESeq2 model and to match the sample sizes used for the other two contrasts, mirroring the binary FLT-versus-GC and unloaded-versus-control designs of the spaceflight and disuse atrophy datasets so that all three conditions could be compared using a structurally identical Wald-statistic framework. A continuous or trend-based model (for example, treating age as a numeric covariate and testing for a monotonic effect across all six timepoints) would make fuller use of the available time-course and could reveal a graded onset of the sarcopenia transcriptional program, but it would not yield a single per-gene Wald statistic directly comparable in form to the binary contrasts used for spaceflight and disuse atrophy. This binary-versus-continuous trade-off is a limitation of the current design and is noted in the Limitations subsection; modeling sarcopenia progression continuously across the full age range is identified as a direction for future work. Disuse atrophy: GSE273092 [25] provided RNA-seq of female C57BL/6 gastrocnemius muscle from a hindlimb unloading paradigm. Analysis was restricted to the wild-type (flox/flox) background and contrasted 10-day unloaded animals (n = 3) against weight-bearing controls (n = 3), so the comparison mirrored the binary spaceflight contrast.

These three datasets were selected because they are publicly available mouse skeletal-muscle RNA-seq datasets that each directly represent one of the three atrophy stressors of primary interest to this study (spaceflight, aging-associated sarcopenia, and disuse); each provides raw or pre-quantified count data suitable for a uniform PyDESeq2 re-analysis, rather than only processed fold-change tables; and each includes a clearly defined binary contrast (stressor versus control or young) enabling a Wald-statistic-based comparison structurally comparable across all three conditions. OSD-576/RR-23 was the dataset of origin for this project, and its selection was therefore fixed. GSE145480 and GSE273092 were identified via a targeted search of the NCBI Gene Expression Omnibus for mouse skeletal-muscle RNA-seq studies modeling sarcopenia and hindlimb-unloading disuse, respectively, published within approximately the last five years and with raw counts deposited [26]. This search was not a formal, protocol-driven systematic review (e.g., a multi-database PRISMA-style search with pre-registered inclusion/exclusion criteria), and other eligible datasets meeting similar criteria may exist and were not evaluated; dataset selection was therefore convenience-based rather than exhaustive. This is acknowledged as a limitation affecting the generalizability of the comparative conclusions, and is discussed further in the Limitations subsection.

For each dataset, raw integer gene counts were filtered to retain genes with at least 10 reads across all samples and analyzed with PyDESeq2 (version 0.5.4) using the design group with the group factor encoded as FLT vs GC, old vs young, or unloading vs control [27,28]. PyDESeq2 estimates size factors via the median-of-ratios method, fits a negative binomial GLM with gene-wise dispersion shrinkage, and reports the Wald statistic, log_2_ fold-change, and Benjamini-Hochberg adjusted p-value for each gene. The Wald test statistic (stat) was retained as a signed, quasi-z-scored measure of differential expression suitable as input to TF activity inference. ENSEMBL identifiers were converted to MGI gene symbols using the GSE145480 annotation tables and a back-up mapping from the OSD-576 gene model; duplicate symbols were resolved by retaining the entry with the largest absolute Wald statistic.

### Transcription factor regulon database

Mouse TF-target interactions were downloaded from CollecTRI through the decoupler-py interface (function dc.op.collectri(organism = ’mouse’)) on the academic license tier [20]. The downloaded regulon comprised 43,226 interactions (TF-target edges) covering 1,165 unique TFs and 16,883 unique target genes. Each interaction carried a signed mode of regulation (’mor’ or weight: + 1 for activation, −1 for repression) curated from PubMed evidence or assigned by the CollecTRI default-activation rule. Following the decoupleR ULM convention, the weight column was passed as the  .mor argument.

### TF activity inference

For each of the three contrasts, the per-gene Wald statistics were concatenated into an 18,940 gene × 3 condition matrix. Missing values (genes absent from a particular contrast) were imputed as zero so that absent genes contribute neither to activation nor inhibition. This zero-imputation reflects a simplifying convention rather than a substantive claim about the true expression status of these genes: absence from a given dataset, whether due to low-count filtering, annotation gaps, or platform-specific detection differences, does not necessarily mean the gene is non-differentially-expressed in that condition, only that no Wald statistic was available for it. Zero-imputation was nonetheless considered reasonable here because it is a neutral choice relative to the alternatives; assigning a non-zero value in the absence of any evidence would risk fabricating a direction of effect, and excluding such genes from a TF’s regulon entirely would reduce statistical power for TFs whose target lists differ most between datasets. This choice could still bias NES scores for TFs whose regulons are enriched for genes that are inconsistently detected across the three datasets, systematically pulling their activity scores toward zero in the affected condition rather than reflecting a true absence of regulatory signal, and this is noted as a limitation. The matrix was wrapped in an AnnData object (samples = conditions, features = genes) and scored with decoupler.mt.ulm using a minimum target count of five (t_min_ = 5). The ULM method fits a single-covariate linear regression of the form y_i_ = β_0_ + β_1_·x_i_ + ε separately for each TF, where y_i_ is the Wald statistic of gene i in the input signature and x_i_ is the signed weight of gene i in the regulon of the TF (zero for non-targets). The enrichment score (NES) is the t-value of the slope coefficient β_1_, and p-values are obtained from the two-sided survival function of the Student’s t distribution and adjusted by Benjamini-Hochberg correction across TFs within each condition [18]. After filtering TFs with fewer than five expressed targets, 732 TFs were scored in all three conditions, yielding the TF activity matrix used in all downstream analyses.

### Comparative analysis and classification

TFs were classified into three mutually exclusive activity categories based on whether the absolute NES reached or exceeded 1.5 in 3, 2, or 1 conditions: ‘Pan-Atrophy’ (active in all three), ‘Partial’ (active in exactly two), and ‘Condition-Specific’ (active in exactly one). The |NES| ≥ 1.5 cutoff is an exploratory activity heuristic adopted for classification purposes, not a formal statistical significance criterion. It is applied directly to the ULM enrichment score (NES) rather than to the Benjamini-Hochberg-adjusted p-value derived from it, and TFs are not additionally required to pass an FDR threshold to be classified as Pan-Atrophy, Partial, or Condition-Specific; classification is therefore threshold-based on effect size alone. This choice prioritizes sensitivity for hypothesis generation over strict control of the false discovery rate. Adjusted p-values for all TF-condition scores are reported separately in S1 Table so that readers who prefer a significance-gated definition of activity can apply one directly. TFs not reaching the threshold in any condition were labeled ‘None’ and excluded from category-level statistics. Pearson and Spearman correlations between condition NES vectors were computed across all 732 TFs using SciPy (version 1.13). Hierarchical clustering of the top-50 most-variable TFs (Fig 2) was performed with seaborn clustermap using Euclidean distance and average linkage on rows. The top-50 cutoff was chosen deliberately to balance visual interpretability of the clustermap against adequate coverage of the most condition-discriminating regulators. This cutoff is a visualization convenience rather than a biological boundary, and TFs excluded from the top 50 are not thereby considered non-discriminating; the same caveat applies to the top-8 cutoff used for Fig 6. TFs were ranked by variance across the three condition NES values specifically because high-variance TFs are the regulators that best distinguish the three atrophy contexts from one another. A one-sided Fisher exact test assessed whether the pan-atrophy class was enriched for a manually curated list of canonical atrophy-pathway TFs (FoxO1, FoxO3, FoxO4, FoxO6, NF-κB family, ATF3, ATF4, DDIT3, Trp53, Stat3, Smad2, Smad3, Smad4, FoxK1, FoxK2, MyoD1, Myog) intersected with the 732 TFs in the regulon. This list was assembled from established reviews of the core muscle-atrophy signaling literature (FoxO-ubiquitin ligase signaling, NF-κB, the integrated stress response, TGF-β/Smad signaling, Trp53, and the myogenic regulators MyoD1/Myog [29]), representing the regulatory axes most consistently implicated across fasting, denervation, glucocorticoid, and immobilization models of atrophy prior to this study, rather than being derived after seeing the present results. To assess robustness of this threshold, classifications were repeated across a range of alternative |NES| cutoffs (1.0–2.5; see Results and S3 Table).

**Fig 1:**
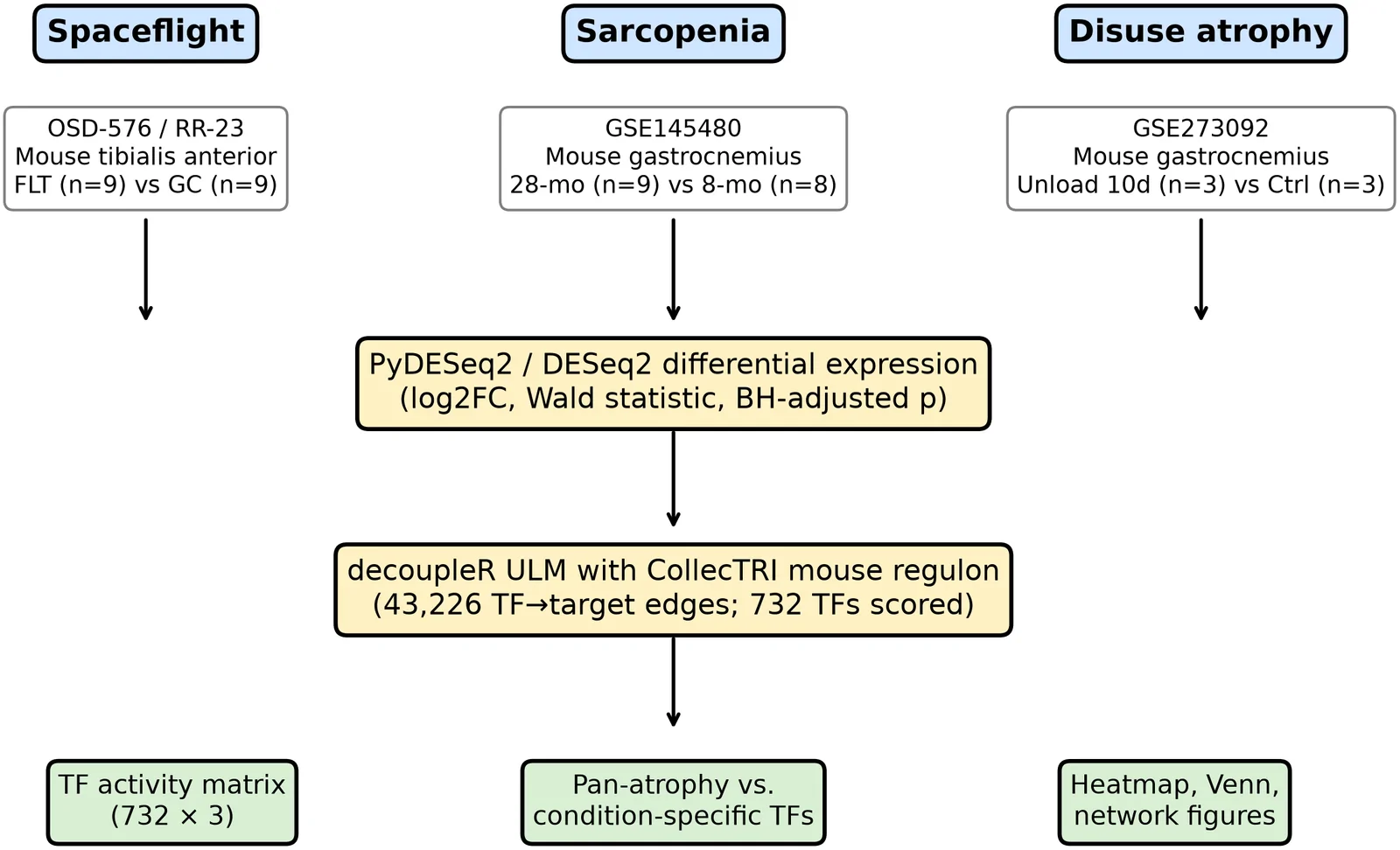
Workflow summary. Three publicly available mouse skeletal-muscle datasets covering spaceflight (OSD-576 / RR-23), aging-associated sarcopenia (GSE145480), and hindlimb-unloading disuse atrophy (GSE273092) were processed with PyDESeq2 to obtain per-gene Wald statistics, then scored against the CollecTRI mouse regulon (43,226 edges, 1,165 TFs) using the decoupleR univariate linear model (ULM) to obtain a 732 TF × 3 condition activity matrix used for clustering, classification, and network analyses.

**Fig 2:**
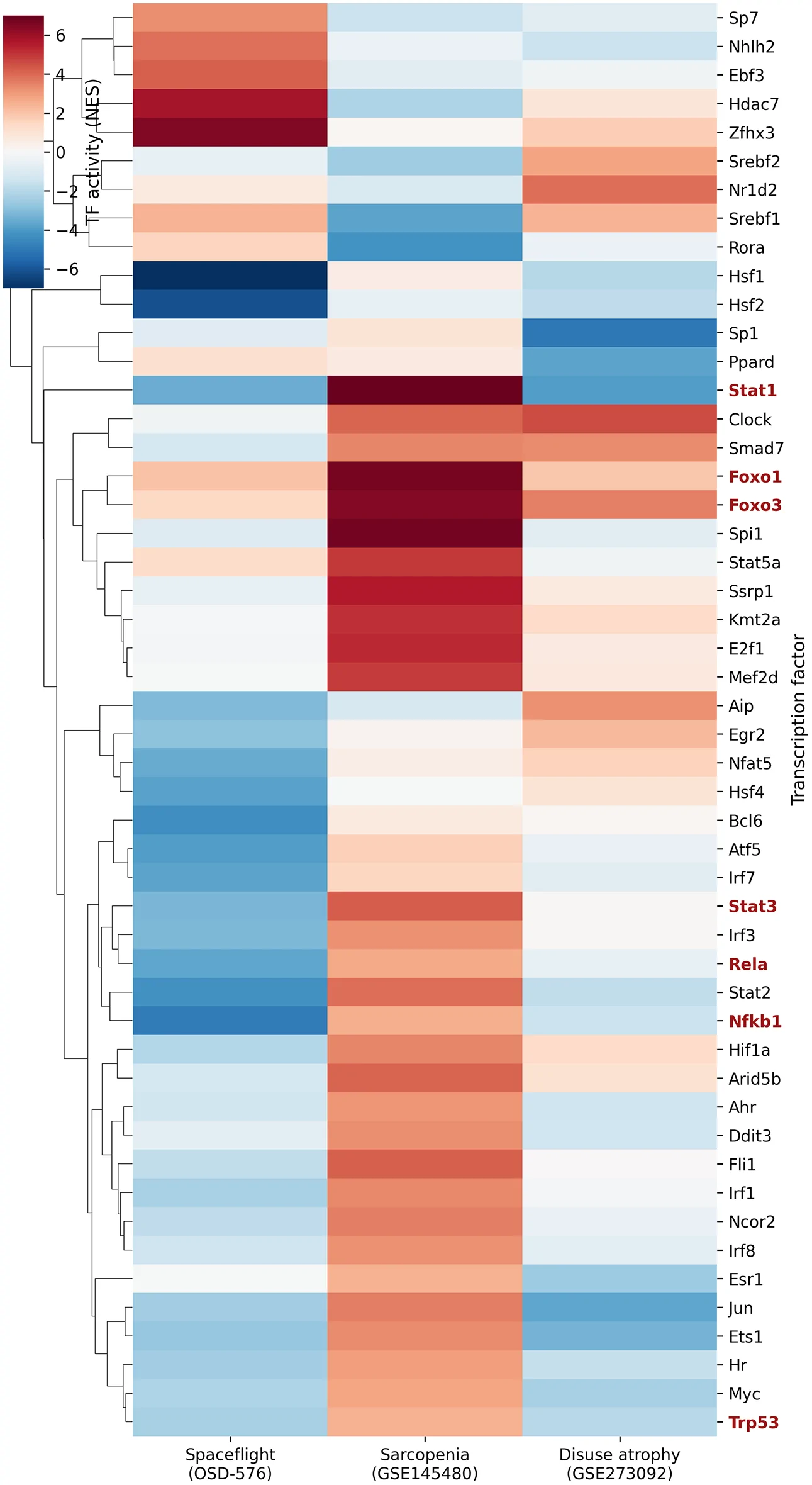
Top 50 most-variable TFs across conditions. Rows are TFs (clustered by Euclidean distance with average linkage), columns are the three conditions. Color scale encodes the ULM normalized enrichment score (NES); red indicates activation and blue indicates repression. Canonical atrophy-pathway TFs (Foxo1, Stat3, Trp53, Nfkb1, Rela, Foxo6) are highlighted in dark red.

### Visualization and software environment

Figures were produced in Python 3.12 using matplotlib (3.9), seaborn (0.13), matplotlib_venn (1.1), networkx (3.3), and adjustText (1.3). Fig 2 used seaborn clustermap with the RdBu_r diverging palette centered at zero. Fig 3 used custom volcano plots with the top 10 TFs per panel selected by the product of |NES| and -log_10_(adj. p) and labeled with adjustText. Fig 4 used matplotlib_venn.venn3 with the active-TF sets (|NES| ≥ 1.5) for each condition. Fig 6 used a force-directed (spring) layout of the directed CollecTRI subgraph spanning the eight concordant pan-atrophy TFs with the largest cumulative |NES|, retaining their eight strongest target edges by absolute weight. The top-8 cutoff for the network visualization was also a deliberate choice intended to keep the resulting graph legible while still capturing the pan-atrophy TFs with the strongest combined activity signal; this criterion is stated explicitly here and mechanically favors TFs with larger, better-annotated CollecTRI regulons independent of true regulatory importance; the resulting network (Fig 6) should be interpreted only as an exploratory visualization of one plausible regulatory architecture among the pan-atrophy set, not as a validated hub map, and is discussed further in the Discussion’s critical-evaluation subsection. All figures were saved as PNG at 300 DPI. Source code and intermediate data are deposited at the GitHub repository linked under Availability of data and materials.

**Fig 3:**
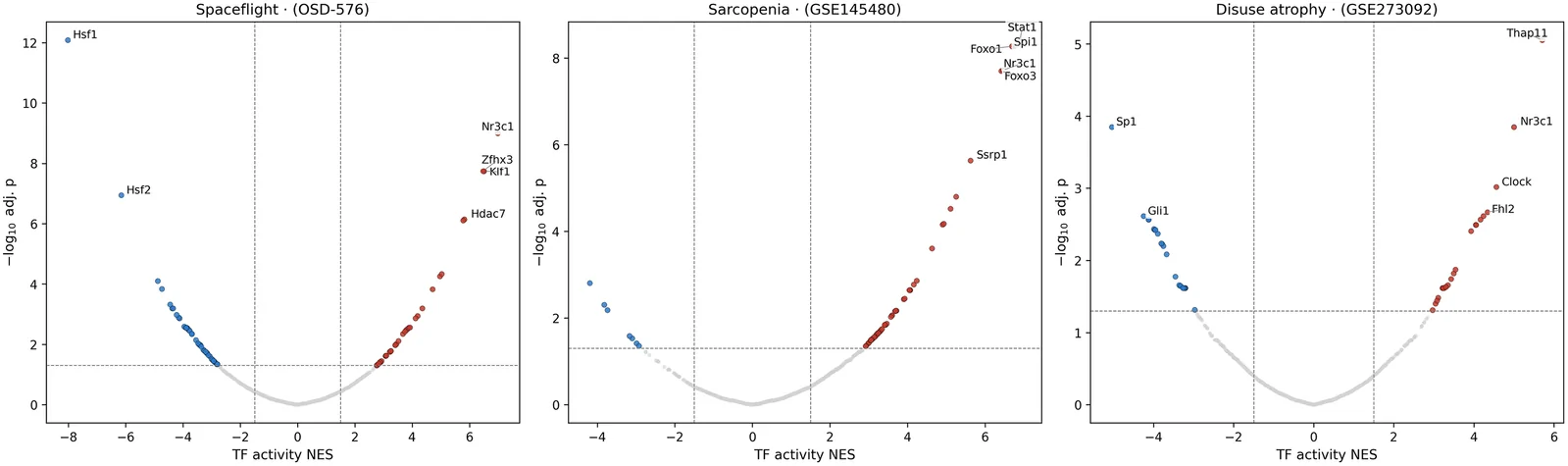
Volcano-style plots of TF activity per condition. X axis: ULM normalized enrichment score (NES). Y axis: -log_10_ of the BH-adjusted ULM p-value. Red and blue points denote TFs above the |NES| ≥ 1.5 and adj. p ≤ 0.05 thresholds (red, activation; blue, repression). The top 10 TFs per condition (by |NES| × -log_10_ p) are labeled.

**Fig 4:**
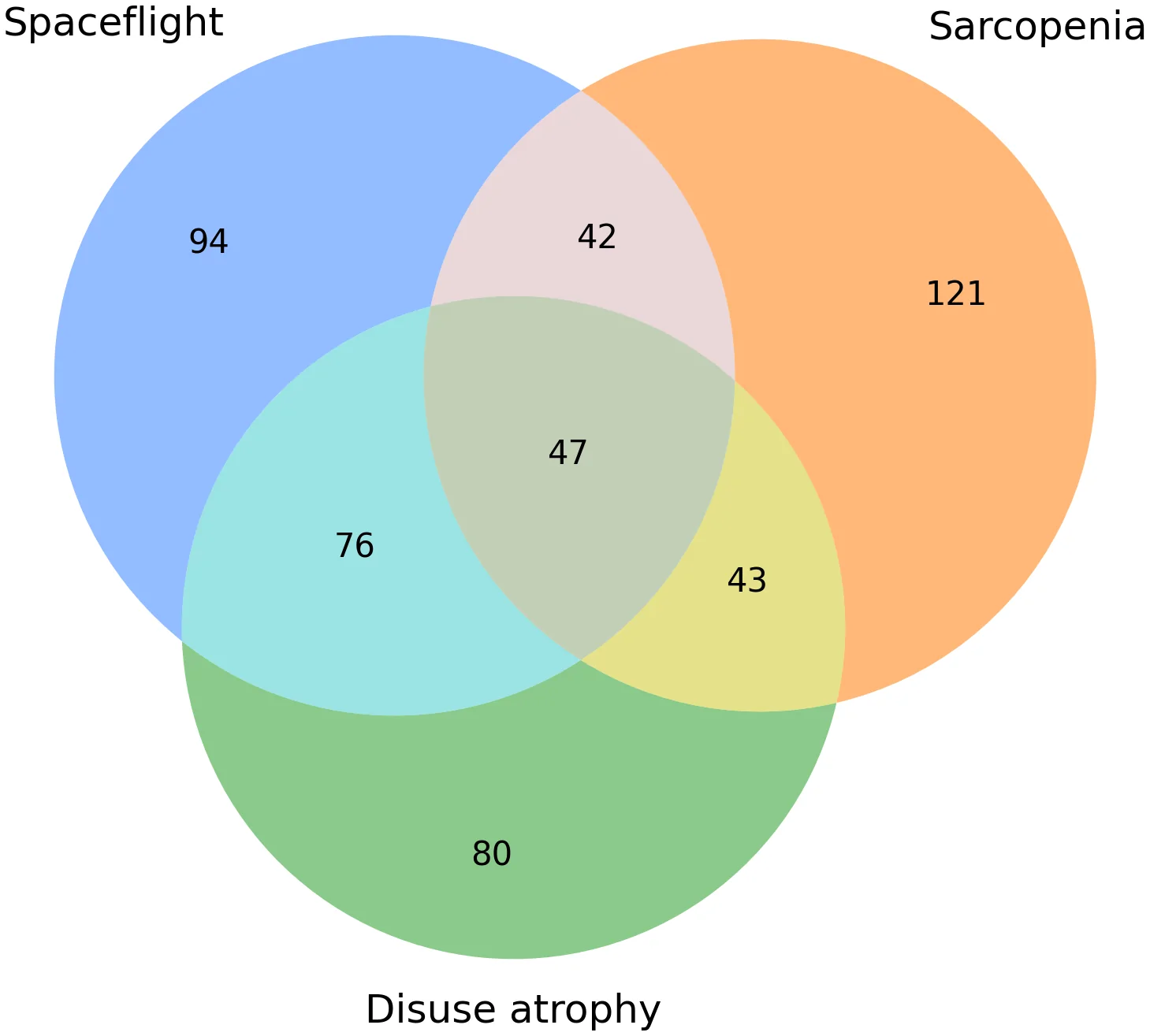
Three-way overlap of active TFs (|NES| ≥ 1.5) across the spaceflight, sarcopenia, and disuse atrophy conditions. The 47 TFs in the central intersection define the Pan-Atrophy class.

## Results

### Dataset overview

Three muscle-atrophy datasets were harmonized at the level of differential expression statistics for entry into the TF activity pipeline (Fig 1). The spaceflight contrast (RR-23, OSD-576) yielded 8,318 genes with finite Wald statistics after low-count filtering, of which 311 reached p_adj_ ≤ 0.05 (the previously reported significant DEG set). The sarcopenia contrast (GSE145480, 28-month vs 8-month gastrocnemius) yielded 17,338 genes with finite statistics and exhibited a markedly stronger global signal, with the top hit Sln showing a Wald statistic of +15.5 (p_adj_ = 7.9 × 10^-50^) and 3,982 genes reaching p_adj_ ≤ 0.05. The disuse atrophy contrast (GSE273092, 10-day hindlimb unloading vs control) yielded 21,239 genes with the top hit Tmem100 at Wald = +13.1 (p_adj_ = 3.9 × 10^-35^) and 1,365 genes at p_adj_ ≤ 0.05. Despite differing tissue (tibialis anterior versus gastrocnemius), magnitude of effect, and stressor mechanism, all three contrasts yielded continuous, well-behaved Wald-statistic distributions suitable for downstream univariate inference.

### TF activity landscape across conditions

After ULM scoring, 732 mouse TFs had ≥ 5 expressed targets and were evaluated in all three conditions, generating 2,196 TF-condition activity scores. The mean absolute NES per condition was 1.23 (spaceflight), 1.18 (sarcopenia), and 1.16 (disuse atrophy). The 75th percentile of |NES| was 1.71, 1.69, and 1.69, respectively, indicating that approximately a quarter of all scored TFs reached the 1.5 threshold in any given condition. The number of TFs reaching NES ≥ 1.5 with adjusted p < 0.05 (a stricter, FDR-gated count reported here for reference only and not used for Pan-Atrophy/Partial/Condition-Specific classification) was 47 in spaceflight, 27 in sarcopenia, and 35 in disuse atrophy. Hierarchical clustering of the 50 most variable TFs across conditions (Fig 2) recovered three broad patterns: a spaceflight-dominant block (Hsf1, Hsf2, Hsf4, Bcl6, Egr2, Aip), a sarcopenia-dominant block enriched for inflammatory and proliferation regulators (Stat1, Spi1, Stat5a, E2f1, Kmt2a, Mef2d), and a third block with concordant activation across conditions (Thap11, Fhl2, Smarca4, Klf1, Nr3c1).

Pearson correlation of TF activity vectors across conditions was highest between spaceflight and disuse atrophy (r = 0.457, 95% CI: 0.398–0.513, p = 4.20 × 10^−39^, n = 732 TFs), intermediate between sarcopenia and disuse atrophy (r = 0.313, 95% CI: 0.246–0.377, p = 4.54 × 10^−18^), and lowest between spaceflight and sarcopenia (r = 0.146, 95% CI: 0.074–0.216, p = 7.44 × 10^−5^). Because these 732 TF activity scores share overlapping CollecTRI regulons, the analytic Fisher z-transformed confidence intervals and p-values above may understate the true uncertainty if treated as fully independent observations. As a complementary check, a label-shuffle permutation test (10,000 permutations) and a TF-level bootstrap (10,000 resamples, which preserves any shared-regulon dependency structure among TFs by construction) were performed using an independently reconstructed CollecTRI mouse regulon closely matched to the primary one (717 of 732 TFs recovered). Permutation testing yielded p ≤ 0.0002 for all three pairwise comparisons, and bootstrap 95% confidence intervals were 0.381–0.512 (spaceflight-disuse), 0.259–0.409 (sarcopenia-disuse), and 0.062–0.220 (spaceflight-sarcopenia), all excluding zero and consistent with the primary analytic estimates. This indicates that the reported correlation pattern is not an artifact of treating TF activity scores as statistically independent. Scatter plots with regression lines and 95% confidence bands for all three pairwise comparisons are shown in S1 Fig. Spearman correlations yielded the same ordering (0.43, 0.35, 0.16). The closer agreement between spaceflight and disuse atrophy is consistent with the well-established mechanical-unloading component of microgravity, while the weaker agreement of either with sarcopenia reflects the additional inflammatory and senescence biology that accompanies aging.

### Pan-atrophy transcriptional regulators

Forty-seven TFs (6.4% of the regulon) reached |NES| ≥ 1.5 in all three conditions and were classified as pan-atrophy (Figs 4, 5; S2 Table). The top pan-atrophy TF by combined absolute activity was the glucocorticoid receptor Nr3c1, which was strongly activated in spaceflight (NES = +6.99), sarcopenia (NES = +6.46), and disuse atrophy (NES = +5.00). Co-activation of Nr3c1 across all three contexts is biologically expected given that elevated glucocorticoid tone is a common upstream driver of skeletal-muscle catabolism in fasting, immobilization, and aging [12]. The FoxO family member Foxo1 showed concordant activation in all three conditions (NES = +2.08, + 6.70, + 1.92), as did the SWI/SNF chromatin remodeller Smarca4 (NES = +5.02, + 2.45, + 3.24) and the stress-response regulators Pml (NES = +4.35, + 1.73, + 2.70) and Ing4. The hypoxia-related TF Epas1 (HIF-2α) was modestly but consistently active (NES = +4.11, + 1.95, + 1.74), suggesting shared activation of hypoxia/oxidative-stress responses despite very different mechanical stimuli. Conversely, the developmental TFs Twist1 (NES = −2.97, −2.16, −2.86) and Ascl1 (NES = −3.40, −2.75, −2.18) were consistently repressed, pointing to a pan-atrophy loss of regenerative/myogenic signaling. Stat1 displayed an unusual sign-discordant pattern, activated in sarcopenia (NES = +6.90) but inhibited in spaceflight (NES = −3.48) and disuse atrophy (NES = −3.89), driven by inflammatory transcript accumulation in aged muscle that is absent in the more acute mechanical models.

**Fig 5:**
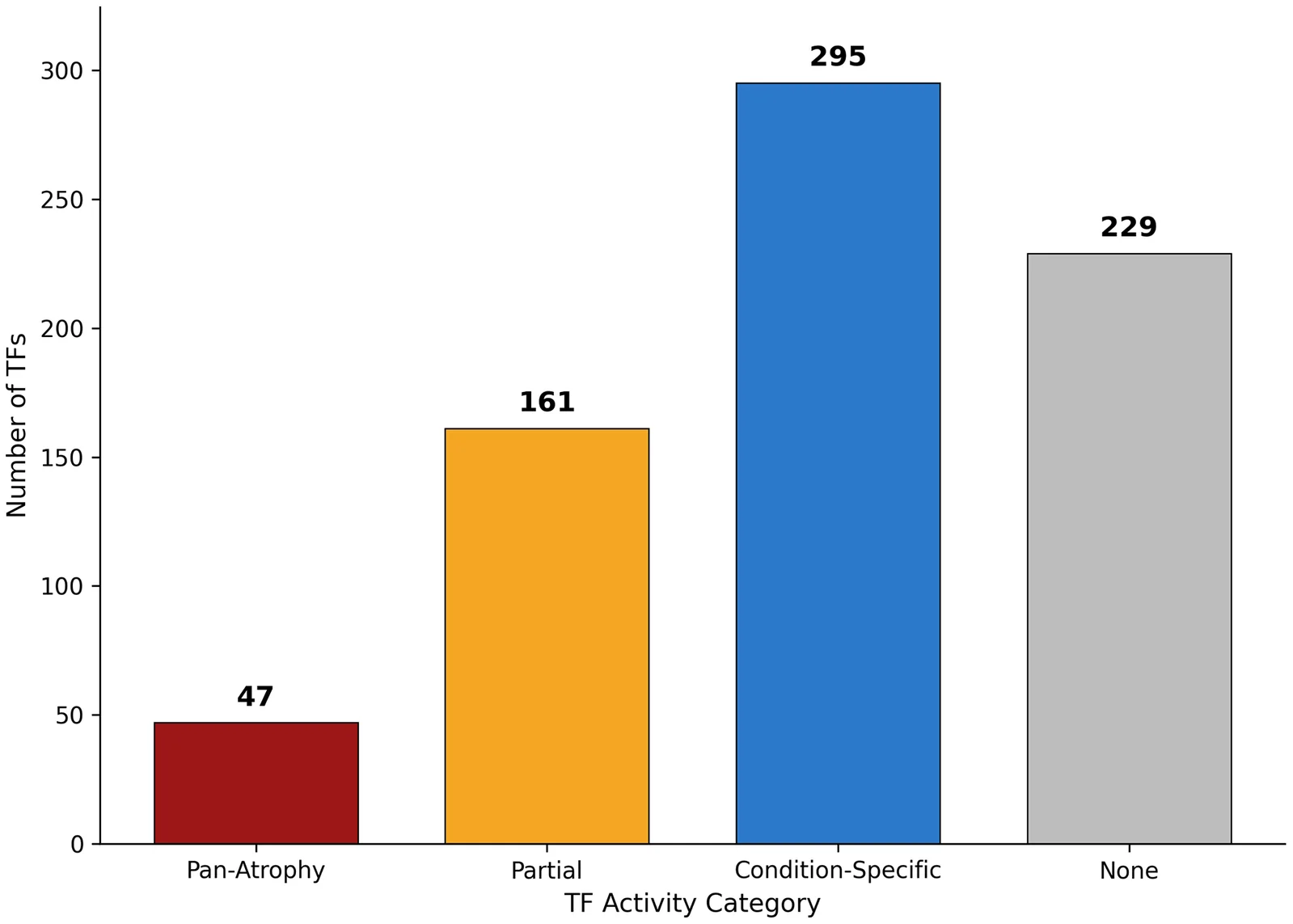
Distribution of the 732 scored TFs across activity categories. Pan-Atrophy: |NES| ≥ 1.5 in all three conditions (n = 47). Partial: |NES| ≥ 1.5 in exactly two conditions (n = 161). Condition-Specific: |NES| ≥ 1.5 in exactly one condition (n = 295). None: not active in any condition under the threshold (n = 229).

Fisher exact testing confirmed that the pan-atrophy class was significantly enriched for canonical atrophy-pathway TFs (FoxO/NF-κB/ATF/Trp53/Smad/IGF axis; 18 TFs in the regulon), with an odds ratio of 4.458 (one-sided p = 2.347 × 10^−2^). Because this canonical TF list was itself curated from the muscle-atrophy signaling literature that motivated the present study’s hypotheses, this enrichment result constitutes a test of internal consistency between the computational Pan-Atrophy classification and prior mechanistic knowledge, rather than an independent validation using an externally defined or blinded gene set. Four canonical atrophy TFs fell in the pan-atrophy class: Foxo1 and Smad3 (concordant) and Nfkb1 and Trp53 (sign-discordant, both repressed in spaceflight and disuse but activated in sarcopenia). Several additional canonical atrophy TFs (e.g., Foxo3, MyoD1) showed strong activity in subsets of conditions but missed the |NES| ≥ 1.5 threshold in one condition and were classified as Partial rather than Pan-Atrophy; inspection of the Partial class confirmed further enrichment for FoxO and ATF family members.

### Robustness of the pan-atrophy classification

To assess sensitivity to the |NES| ≥ 1.5 classification threshold used throughout this study, TF classification was repeated across six thresholds ranging from 1.0 to 2.5 (S3 Table). The proportion of TFs classified as Pan-Atrophy declined smoothly and monotonically with increasing stringency, from 14.3% (105 TFs) at |NES| ≥ 1.0 to 0.4% (3 TFs) at |NES| ≥ 2.5, with Pan-Atrophy sets at each threshold forming perfectly nested subsets of the more permissive sets. Because Pan-Atrophy is defined by an absolute-value cutoff applied uniformly across conditions, this nesting property is an expected mathematical consequence of raising the threshold rather than independent evidence of robustness; any TF that meets a stricter cutoff also meets every more permissive one. What the monotonic, discontinuity-free decline does show is that the Pan-Atrophy classification does not exhibit abrupt or unstable behavior near the primary threshold of 1.5; it does not by itself demonstrate that the identity or ranking of the leading Pan-Atrophy regulators is robust to the choice of cutoff. The glucocorticoid receptor Nr3c1 remained classified as Pan-Atrophy at every threshold tested (1.0–2.5), reflecting its consistently large absolute NES in all three conditions, which is a separate and more informative observation than the nesting property itself. Smarca4 and Ing4 remained Pan-Atrophy through |NES| ≥ 2.0, and Foxo1 through |NES| ≥ 1.75, while Pml was the least robust of the five core regulators, dropping out of the Pan-Atrophy class above |NES| ≥ 1.5.

### Condition-specific TF activity

Two hundred and ninety-five TFs (40.3%) were active in only one condition (Figs 4, 5). Spaceflight-associated TFs (relative to the gastrocnemius comparators) were dominated by repression of the heat shock factor family (Hsf1 NES = −8.02, + 0.58, −2.00; Hsf2 NES = −6.15, −0.57, −1.76; Hsf4 NES = −3.79, 0.00, 0.96), repression of the inflammatory TF Bcl6 (NES = −4.37) and the proliferation-associated Nfyc (NES = −4.13), and activation of the histone deacetylase Hdac7 (NES = +5.82, −2.14, + 0.92) and the muscle-specifying TF Sox6 (NES = +3.92). The sweeping downregulation of HSF activity is a striking spaceflight-enriched signature (relative to the gastrocnemius comparators) consistent with prior reports that spaceflight elicits a blunted heat-shock response and reduced proteostatic capacity in skeletal muscle [8]. Activation of Hdac7, a class IIa HDAC that represses MEF2 target genes upon nuclear retention, suggests a spaceflight-enriched shift toward repression of myogenic structural transcription partially decoupled from the FoxO axis.

Sarcopenia-specific TF activity was dominated by activation of inflammatory and lymphoid-affiliated regulators not prominent in the acute models. Spi1/PU.1 (NES = +6.75), Stat5a (NES = +4.93), and Irf2 (NES = +3.68) were all uniquely active in aged muscle, consistent with the well-described myeloid infiltration and low-grade chronic inflammation that accompanies sarcopenia. The histone methyltransferase Kmt2a (MLL1; NES = +5.11), the chromatin co-factor Ssrp1 (NES = +5.62), the cell-cycle regulator E2f1 (NES = +5.25), and the muscle-restricted MEF2 family member Mef2d (NES = +4.90) defined a sarcopenia-specific chromatin-remodeling and proliferation block. Rora, a circadian-clock TF whose loss has been linked to muscle dysfunction, was uniquely repressed (NES = −4.19), potentially reflecting age-associated circadian disruption in skeletal muscle.

Disuse atrophy-specific TFs included strong activation of the carbohydrate-responsive lipogenic regulator Mlxipl/ChREBP (NES = +4.23) and the developmental TF Ovol1 (NES = +4.16), alongside repression of Ppard (PPARδ; NES = −3.76) and the proliferation-associated TFs Sp1 (NES = −5.04), Prrx1 (NES = −3.25), Hdgf (NES = −3.36), and Myb (NES = −3.20). The Ppard signature is consistent with the well-known shift away from oxidative fiber programs during 10-day hindlimb unloading and parallels reduced fatty-acid utilization phenotypes reported in the original GSE273092 study. Mlxipl activation is a less anticipated finding and may reflect the elevated glycolytic-fiber transcription that follows acute unloading. The volcano plots (Fig 3) summarize these distributions for each condition and highlight the top 10 TFs per condition by combining effect size and significance.

### Target-gene network analysis of pan-atrophy TFs

To examine the regulatory targets linking pan-atrophy TFs to downstream muscle phenotypes, a directed subgraph was extracted from CollecTRI. This subgraph focused on the eight concordant pan-atrophy TFs with the largest cumulative absolute |NES|: Nr3c1, Thap11, Klf1, Fhl2, Smarca4, Foxo1, Ing4, and Gli1. For each TF, only the eight strongest target interactions, determined by absolute weight, were retained. The resulting network (Fig 6) contained 8 source nodes, 53 unique target nodes, and 61 directed edges. Among these edges, 53 (86.9%) encode activation and 8 (13.1%) encode repression. Nr3c1 and Foxo1 shared activating edges to apoptosis-related targets including Bcl2 and Tnfsf10/TRAIL, consistent with their cooperative role in stress-induced muscle wasting. Foxo1 additionally activated the ubiquitin-proteasome and autophagy machinery via Cdkn1b, Igfbp1, Hbb-b1/Hbb-bh1, and Bnip3-related targets that are well-known atrogene candidates [10]. Smarca4 connected to a chromatin-remodeling and cell-cycle target panel (Ccnd1, Cdkn2a, Tcf3) and Klf1 to terminal differentiation targets, illustrating that the pan-atrophy program is not exclusively proteolytic but also includes regulators of fiber identity and proliferation. Because both the eight-TF hub selection and the retained edges reflect an arbitrary top-N cutoff applied to cumulative absolute NES and edge weight rather than a statistical test of hub centrality, this network is presented as a preliminary, hypothesis-generating visualization. It suggests that a small number of regulators, in particular Nr3c1 and Foxo1, are candidates for coordinating multiple downstream atrophy effectors, but the analysis does not establish hub status, causal influence, or the completeness of this regulatory picture. This observation is consistent with, but does not itself provide new evidence for, prior pharmacological interest in antagonizing glucocorticoid signaling for muscle preservation.

**Fig 6:**
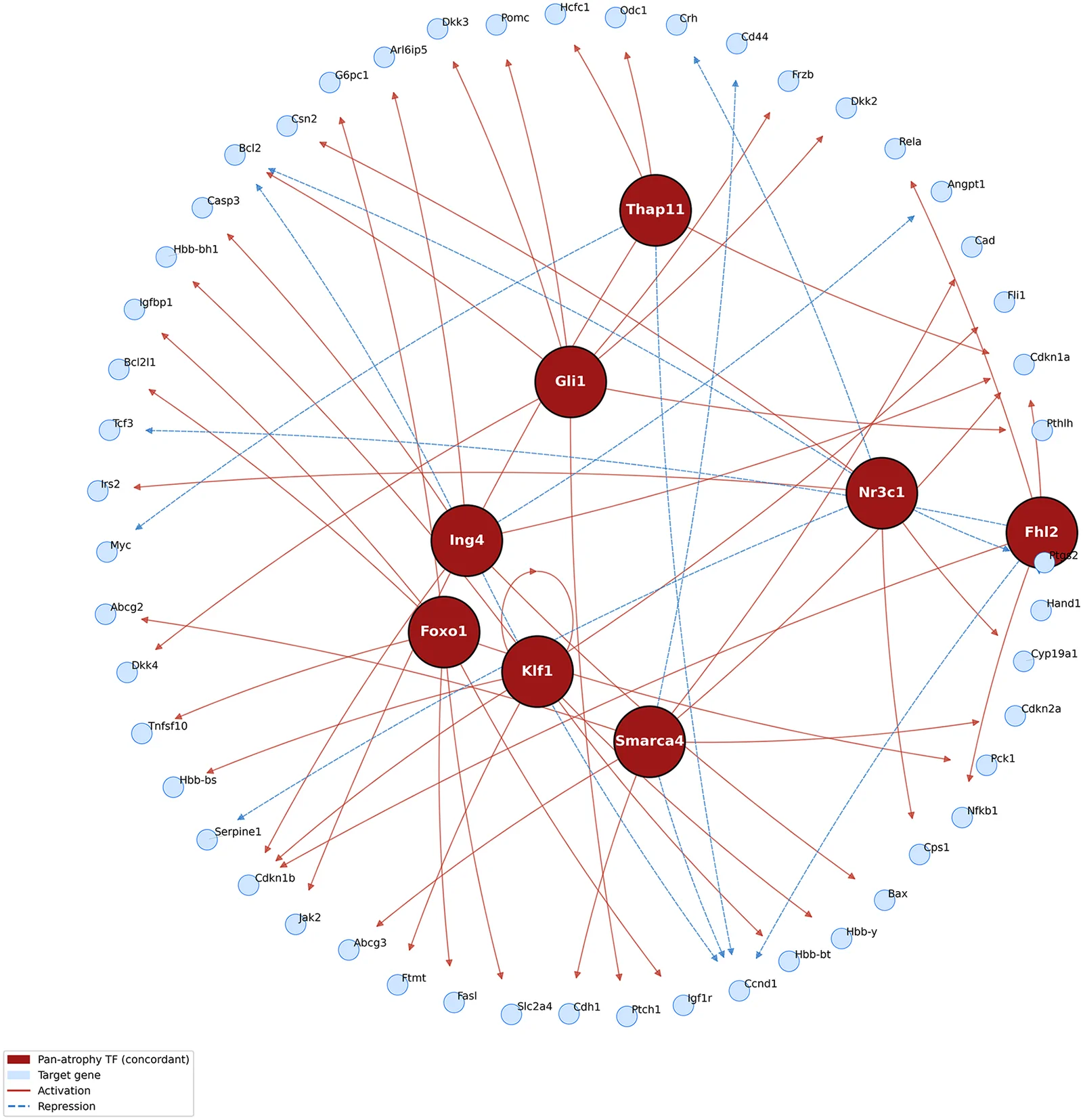
CollecTRI regulatory network of leading pan-atrophy TFs. The eight concordant pan-atrophy TFs with the largest cumulative |NES| (Nr3c1, Thap11, Klf1, Fhl2, Smarca4, Foxo1, Ing4, Gli1) are connected to their eight strongest target genes (light-blue nodes) by activating (solid red) or repressive (dashed blue) edges. The network is visualized with a force-directed (spring) layout. Sign-discordant pan-atrophy TFs (Stat1, Stat2, Ets1, Jun, Fos, Hr, Myc, Nfkb1, Trp53, Trp63, E2f5, Xbp1, Zfp395, Srebf1, Foxe1) are excluded from this network visualization. This visualization reflects an arbitrary top-8/top-edge selection and should be interpreted as exploratory rather than as evidence of causal hub structure.

## Discussion

### Validation of the analytical framework

By moving beyond gene-level overlap to the level of inferred TF activity, a small but biologically coherent pan-atrophy regulatory program was discovered, which spans spaceflight, aging-associated sarcopenia, and hindlimb-unloading disuse atrophy. Of 732 mouse TFs scored, 47 (6.4%) reached |NES| ≥ 1.5 in all three conditions, with the glucocorticoid receptor Nr3c1 emerging as the single most consistent and most strongly activated regulator, followed by Foxo1, Smarca4, Klf1, Thap11, and Fhl2. The recovery of Nr3c1 and Foxo1 at the top of the pan-atrophy list constitutes an important validation of the analytical framework: both factors are established master regulators of catabolic transcription in skeletal muscle [2,10,12]. Identifying them by an entirely orthogonal network-statistical approach, using only target-gene Wald statistics and a literature-curated regulon, demonstrates that ULM scoring of CollecTRI is sensitive to canonical biology even when the underlying DEG overlap between conditions is modest.

### Novel pan-atrophy regulators and sign-discordant TFs

Beyond the canonical FoxO/glucocorticoid axis, several pan-atrophy TFs identified here are less frequently emphasized in muscle-wasting reviews and offer hypotheses for further mechanistic study. The activation of Pml across all three conditions echoes the role of PML nuclear bodies in oxidative-stress and senescence responses, both of which are activated in spaceflight, aging, and unloading. The concordant activation of the SWI/SNF catalytic subunit Smarca4 implies a shared chromatin-remodelling component to the atrophy transcriptome, complementary to the well-known histone-deacetylase axis. Persistent repression of the developmental TFs Twist1 and Ascl1, together with the Hedgehog effector Gli1, points to a pan-atrophy contraction of myogenic and regenerative programs. The stress-response TF Stat1, while pan-atrophy by absolute |NES|, displays a sign-discordant pattern (activated in sarcopenia, repressed in spaceflight and disuse), highlighting that the inflammatory burden of aging operates qualitatively differently from acute mechanical unloading. Fifteen pan-atrophy TFs displayed sign-discordant patterns. The largest group (Ets1, Jun, Fos, Hr, Myc, Stat1, Stat2, Nfkb1, Trp53, Trp63, E2f5, Xbp1, Zfp395) was repressed in spaceflight and disuse but activated in sarcopenia, consistent with the inflammatory and stress-response transcription that accumulates in aged muscle but is less prominent in acute mechanical unloading. Two TFs showed the opposite pattern: Srebf1 and Foxe1 were activated in spaceflight and disuse but repressed in sarcopenia, potentially reflecting metabolic shifts during acute unloading that reverse with chronic aging.

### Spaceflight-enriched regulatory signatures

Spaceflight-enriched TF activity (relative to the gastrocnemius comparators) offers an interpretive lens on what distinguishes the tibialis anterior spaceflight signature from the gastrocnemius-based sarcopenia and disuse comparators at the regulatory level. The striking inactivation of the heat shock factor family (Hsf1, Hsf2, Hsf4) in spaceflight is consistent with reports that astronauts and rodent ISS samples display blunted proteostatic responses despite comparable mechanical-unloading components, and may relate to the compounded cellular stresses (radiation, fluid shifts, circadian disruption) experienced in orbit; however, Hsf2 also met the activity threshold in disuse atrophy, indicating this signature is not strictly spaceflight-enriched. Activation of the muscle-restricted Hdac7 was spaceflight-enriched (relative to the gastrocnemius comparators) and may underlie spaceflight-associated repression of MEF2-target structural genes. By contrast, sarcopenia showed enrichment of myeloid (Spi1/PU.1) and STAT5/IRF inflammatory TFs, supporting the view that aging muscle wasting is driven, at least in part, by chronic low-grade inflammation distinct from acute atrophy. Disuse atrophy showed enrichment of the lipogenic regulator Mlxipl and repression of Ppard, fitting an oxidative-to-glycolytic fiber shift that has been documented physiologically but not previously linked to these TFs at the activity level; this finding is hypothesis-generating given the n = 3 vs n = 3 sample size limitation.

### Critical evaluation of findings and alternative explanations

Several of the findings emphasized above admit plausible alternative explanations that merit explicit consideration. First, the prominence of Nr3c1 and Foxo1 at the top of the Pan-Atrophy list could partly reflect regulon size and annotation depth rather than true biological convergence: TFs with larger, better-curated target-gene sets in CollecTRI are statistically more likely to achieve extreme NES values under the ULM framework, independent of whether their true regulatory role differs from less-annotated TFs. This cannot be fully excluded as a contributing factor to the ranking observed, although the concordant direction of activation across three independently generated datasets argues against pure annotation artifact.

Second, as noted in Results, the Fisher exact test reflects internal consistency rather than independent validation, since the canonical TF list was drawn from the literature that motivated the study’s hypotheses.

Third, the correlation structure observed (spaceflight-disuse → sarcopenia-disuse → spaceflight-sarcopenia) is consistent with the proposed mechanical-unloading interpretation, but an equally parsimonious alternative is that it reflects residual tissue-of-origin similarity, since the sarcopenia and disuse datasets both sampled gastrocnemius while spaceflight sampled tibialis anterior. If tissue identity were the dominant driver, however, the sarcopenia-disuse pair (same tissue) would be expected to show the highest correlation, which it did not; the spaceflight-disuse pair (different tissues, shared mechanical-unloading biology) instead showed the strongest agreement, which is more consistent with the biological interpretation than with a pure tissue-confound explanation. This argument alone is not conclusive, however: it relies on a single directional prediction (same-tissue pairs should correlate most strongly under a tissue-driven model) rather than a direct test that isolates tissue-of-origin effects from biological convergence, and a partial tissue contribution to the observed correlation structure cannot be ruled out. A dataset with matched tissue across all three atrophy contexts, or an explicit fiber-type-composition covariate, would be needed to fully resolve this ambiguity.

Fourth, the sign-discordant activity of several pan-atrophy TFs (Stat1, Jun, Fos, Nfkb1, and others) in sarcopenia relative to spaceflight and disuse atrophy may reflect a genuine biological difference in inflammatory signaling with age, but could equally arise from shifts in tissue cellular composition (e.g., immune cell infiltration in aged muscle) rather than a cell-intrinsic change in TF activity within myofibers; bulk RNA-seq cannot distinguish these possibilities, and single-cell or spatial data would be needed to resolve this ambiguity.

Finally, the hub selection for the regulatory network analysis (Fig 6) was based on cumulative absolute NES across the eight leading pan-atrophy TFs, a criterion that, like the Pan-Atrophy classification itself, may favor TFs with large regulons; the resulting network should therefore be read strictly as a hypothesis-generating visualization, not as evidence of causal hub structure, regulatory hierarchy, or completeness.

### Limitations

The analysis has several limitations. First, all three datasets are from mouse skeletal muscle, but they sample different muscles (tibialis anterior versus gastrocnemius), different sexes within datasets, and different ages of baseline animals; tissue-of-origin differences between tibialis anterior (mixed slow/fast) and gastrocnemius (predominantly fast) almost certainly contribute to the moderate Pearson correlations observed and bias the Pan-Atrophy class toward TFs robust to fiber-type composition. Second, the disuse-atrophy contrast was based on n = 3 versus n = 3 wild-type animals from GSE273092, which limits statistical power and produces wider confidence intervals on the ULM NES; replication in a larger unloading cohort, such as bed-rest human studies, would strengthen condition-specific calls. Third, ULM and CollecTRI are blind to post-translational regulation; activation of a TF’s target transcripts is interpreted as activation of the TF itself, but in principle could reflect derepression by a co-factor, altered chromatin accessibility, or competing regulators sharing the same targets. Fourth, the |NES| ≥ 1.5 threshold is a convention that balances sensitivity and specificity but is not adaptive to the sample size of each contrast; spaceflight, with the smallest underlying sample, is somewhat less likely to clear the threshold than the larger sarcopenia cohort, biasing the Partial class against spaceflight. Fifth, no explicit batch-effect correction was applied across the three datasets, which were generated by different laboratories using different sequencing platforms, library preparation protocols, and processing pipelines. Because each dataset was analyzed independently with PyDESeq2 before TF activity inference, condition-specific technical variation is largely absorbed within each contrast’s own normalization and dispersion estimation rather than propagated across datasets; however, it cannot be ruled out that residual batch-driven differences in gene detection sensitivity or library complexity contribute to the moderate (rather than strong) inter-condition correlations observed, particularly for the spaceflight-sarcopenia comparison. Sixth, comparing Wald statistics across three independently fitted DESeq2 models assumes these values are approximately comparable in scale across datasets. Because the Wald statistic is a signed, variance-standardized measure of differential expression (effectively a z-score under the null), and because decoupleR’s ULM method further standardizes this signal by regressing it against a common regulon rather than comparing raw magnitudes directly, this approach is more robust to between-dataset scale differences than direct comparison of Wald statistics or fold-changes alone would be. Nonetheless, formal validation of Wald-statistic comparability across independently processed RNA-seq datasets remains an open methodological question in the field, and the conducted TF-activity comparisons should be interpreted as relative rankings within the regulon rather than absolute cross-study effect sizes. This argument for comparability is currently narrative rather than empirically tested. As a complementary check, TF classification was re-derived after converting each dataset’s per-gene Wald statistics to within-dataset rank-based z-scores prior to ULM scoring, removing any dependence on the absolute scale of the Wald statistic. The overall ranking of TFs by combined absolute NES was strongly correlated between the raw-Wald and rank-based analyses (Spearman ρ = 0.740, p = 4.57 × 10^−128^, n = 732 TFs), indicating that relative TF activity rankings are largely robust to this choice of statistic. The discrete Pan-Atrophy classification was more sensitive to the transformation, however: only 27 TFs were classified as Pan-Atrophy under the rank-based analysis compared to 47 under the primary raw-Wald analysis, with a Jaccard overlap of 0.396 between the two sets (Jaccard = 0.343 when comparing the top 47 TFs by combined activity under each approach). This indicates that while continuous TF-activity ranking is comparatively stable across this cross-dataset statistic transformation, the discrete threshold-based classification used throughout this study is more sensitive to the specific choice of input statistic, consistent with its characterization as an exploratory rather than confirmatory heuristic (see Methods, “Comparative analysis and classification”). Seventh, dataset selection (see Methods) was pragmatic and availability-based rather than the product of a systematic, protocol-driven search across multiple databases; other publicly available datasets modeling these or related atrophy stressors may exist and were not considered, which limits confidence that the three datasets analyzed here are representative of the broader universe of muscle-atrophy transcriptomic studies. Eighth, the sarcopenia contrast used only the oldest and youngest timepoints available in GSE145480 (28 versus 8 months), discarding the four intermediate ages (18, 22, 24, and 26 months) in order to preserve a binary contrast structure comparable across all three conditions. This maximizes power and comparability but does not characterize the trajectory or rate of onset of the sarcopenia transcriptional program, which a continuous, age-as-covariate model could address in future work.Ninth, zero-imputation of genes absent from a given dataset assumes these genes contribute no activating or repressing signal to TF scoring in that condition, but absence from a dataset does not necessarily indicate zero true differential expression; it may instead reflect low sequencing depth, incomplete gene annotation, or platform-specific filtering. This could disproportionately affect NES scores for TFs whose CollecTRI regulons are enriched for genes inconsistently detected across the three datasets, and is a limitation of the current imputation strategy. Tenth, the standard Pearson confidence intervals and p-values reported for TF activity correlations assume independent observations across the 732 scored TFs, but CollecTRI regulons overlap substantially, so these intervals should be interpreted as approximate. A permutation- and bootstrap-based check (Results) using an independently reconstructed regulon supports the same qualitative conclusions, but exact numerical agreement with the primary analytic statistics was not expected given the use of a closely matched, though not identical, regulon reconstruction.

### Strengths and future directions

Despite these limitations, the framework has clear strengths. By using a literature-curated regulon and a univariate, interpretation-friendly statistic, descriptive DEG-overlap analysis is moved towards mechanistic regulatory hypotheses that are directly testable. The pan-atrophy TFs identified, particularly Nr3c1 and Foxo1, are pharmacologically tractable and have published small-molecule modulators (e.g. SARMs that uncouple glucocorticoid signaling from muscle catabolism, or selective Akt activators that phosphorylate and inactivate FoxO factors) whose effects could now be evaluated against this triplet of atrophy contexts. Future work should extend the approach to single-cell datasets to resolve TF activity at the level of muscle-fiber and immune subtypes, incorporate human muscle-biopsy datasets from immobilization and bed-rest studies for cross-species validation, and integrate ATAC-seq accessibility data to discriminate TF activity from cofactor-mediated derepression. Within space biology specifically, the spaceflight-enriched signatures (HSF inactivation, Hdac7 activation) relative to the gastrocnemius comparators are testable countermeasure targets and motivate hypothesis-driven studies in upcoming Rodent Research missions.

## Conclusions

Comparative TF-activity inference across spaceflight, sarcopenia, and disuse atrophy reveals a 47-member pan-atrophy regulatory program led by Nr3c1, Foxo1, Smarca4, Klf1, Pml, Thap11, and Fhl2 (with Stat1, Stat2, Ets1, Jun, Fos, Hr, Myc, Nfkb1, Trp53, Trp63, E2f5, Xbp1, Zfp395, Srebf1, and Foxe1, fifteen TFs in total, meeting the absolute threshold but showing sign-discordant activity patterns across conditions), accompanied by much larger pools of partial (n = 161) and condition-specific (n = 295) TFs that encode the stressor of origin. The pan-atrophy core encompasses both classical proteolytic regulators and less-emphasized chromatin-remodeling, oxidative-stress, and regenerative-loss factors, while spaceflight is distinguished by heat-shock-factor inactivation and Hdac7 activation, sarcopenia by myeloid/STAT inflammatory activation, and disuse atrophy by Mlxipl/Ppard metabolic re-wiring. These findings narrow the candidate regulator list relevant to muscle-mass preservation in clinical and space-medicine contexts, flag regulators disproportionately active in the spaceflight comparison as hypotheses for further study, and demonstrate the added mechanistic resolution of TF-level comparative analysis over conventional DEG comparison. It is important to consider that these are computationally inferred candidates requiring experimental validation, not established therapeutic targets.

## Supporting information

S1 Fig Pairwise scatter plots of TF activity (NES) across conditions. Each panel shows the relationship between normalized enrichment scores (NES) for all 732 scored transcription factors in one pair of conditions, with an ordinary least-squares regression line and shaded 95% confidence band. Pearson r, 95% confidence interval (Fisher z-transformed), and p-value are reported above each panel.
(PNG)

S1 Table Full transcription factor activity matrix across all three conditions. Normalized enrichment scores (NES) and Benjamini-Hochberg adjusted p-values (p_adj_) from decoupleR ULM scoring are reported for all 732 transcription factors evaluated in the spaceflight, sarcopenia, and disuse atrophy contrasts. Each TF is assigned to one of four activity categories (Pan-Atrophy, Partial, Condition-Specific, None) based on the number of conditions in which |NES| ≥ 1.5, with the direction of significant activity in each condition (sig_directions) also reported.
(CSV)

S2 Table Pan-Atrophy transcription factor list. The 47 transcription factors classified as Pan-Atrophy (|NES| ≥ 1.5 in all three conditions) are listed with their NES values in spaceflight, sarcopenia, and disuse atrophy; the minimum adjusted p-value across conditions (p_adjmin_); the direction of activity in each condition (sig_directions); and combined activity metrics (mean and summed absolute NES), ranked by summed absolute NES.
(CSV)

S3 Table Sensitivity of TF classification to the |NES| activity threshold. Category counts (Pan-Atrophy, Partial, Condition-Specific, None), percentage of TFs classified as Pan-Atrophy, and overlap (raw count and Jaccard index) of the Pan-Atrophy TF set relative to the primary threshold (|NES| ≥ 1.5) are reported across six threshold values (1.0–2.5).
(CSV)
